# Diversity and Functional Roles of Microorganisms in Anatolian Black Pine Cone Vinegar Fermentation

**DOI:** 10.1002/fsn3.70155

**Published:** 2025-04-11

**Authors:** Duygu Alp‐Baltakesmez, Pelin Ertürkmen, Özcan Bulantekin

**Affiliations:** ^1^ Department of Gastronomy and Culinary Arts, School of Tourism and Hospitality Management Ardahan University Ardahan Turkey; ^2^ Department of Food Processing, Vocational School of Burdur Food, Agriculture and Livestock BurdurMehmet Akif Ersoy University Burdur Turkey; ^3^ Department of Cookery, Agri, Higher Vocational School of Doğubayazıt Ahmed‐i Hani Agri Ibrahim Cecen University Ağrı Turkey

**Keywords:** Anatolian black pine cone, antimicrobial activity, bioactive components, microbiota, vinegar

## Abstract

The parts of some pine species are a rich source of bioactive compounds that can be used in various food products. The current work, the physicochemical, bioactive, antimicrobial, sensory, and aromatic properties of traditional vinegar produced from Anatolian Black Pine Cones from different provinces of Turkey were determined, as well as the cultivable microbial diversity and metagenomic analysis. The total phenolic content of the vinegars ranged from 163.88 to 174.79 mg GAE/L. Antioxidant activity, measured via DPPH and ABTS assays, varied among the samples. CnB vinegar, made from Burdur province cones, stood out for its bioactive compounds, including terpenes, acetic acid, ascorbic acid, and the highest α‐terpineol content (3.13%). CnB also exhibited the strongest antimicrobial activity, with the largest inhibition zone (44.91 mm) against *E. coli* type A, while CnM showed the lowest activity. Sensory evaluations favored CnB for its balanced flavor, while CnV was criticized for excessive sharpness, and CnM was deemed too mild. The bacterial microbiome of CnB was predominantly composed of acetic acid bacteria, with an average concentration of 7.36 log CFU/mL in the enumeration of culturable microorganisms. The dominant bacterial taxa at the phyla level included *Proteobacteria* (72.296%), *Firmicutes* (22.062%), *Bacteroidota* (3.665%), followed by *Acetobacteraceae* (71.47%), *Clostridia* (13.187%), *Bacilli* (5.066%), Bacteroidetes (3.665%), and *C. negativicutes* (3.737%) at the phylum level. The fungal microbiome was mainly represented by *Ascomycota* (78.717%) and *Eukaryota Incertae sedis* (15.840%). The findings demonstrate that pine cone vinegar can be employed in a multitude of applications, including food preservation and health promotion.

## Graphical abstract

The pine cone vinegar has been analyzed for its microbial community using cultural methods and metagenomic analysis, as well as its aroma components, total phenolic content, antioxidant capacity, and organic acids. The results provide valuable information on the sensory properties, aroma volatiles, antimicrobial effect, and relationship with the microbial community of vinegars produced from Anatolian Black Pine cones.

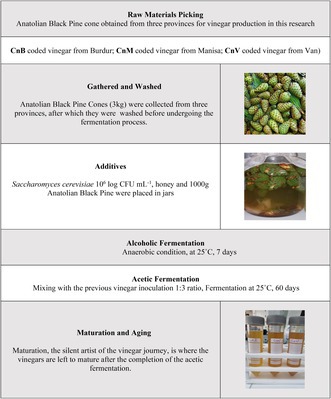

## 1 Introduction

Pinus species are hard and two‐conifer pine trees (Ioannidis et al. 2017; Gamli 2022), *Pinus nigra* ( *Pinus nigra* Arnold. subsp. *pallasiana* (Lamb.) Halmboe) is also known as “Anatolian Black Pine” in Turkey. It is a species that grows widely in the Mediterranean region and Western and Northern Anatolia (Carus and Çatal 2007; Negiz et al. 2019). The cones of *Pinus nigra* spp. have been reported to contain various amino acids (glutamic acid, glycine, histidine, lysine, and threonine) and monosaccharides (glucose, mannose, xylose, and galactose) in addition to steroids, flavonoids, terpenoids and procyanidins in their extracts (Ren et al. 2012). The antifungal, antibacterial and wound‐healing activities of these compounds have been demonstrated in the studies conducted by Smith et al. (2005), Lantto et al. (2009) and Süntar et al. (2012). It is reported that extracts obtained from pine cones have very good antioxidant activity and this property is related to their physicochemical properties (Zou et al. 2013; Xu et al. 2016; Gamli 2022). Studies have demonstrated that various parts of the pine plant, including leaves and cones, have been traditionally used for medicinal and culinary purposes for many years. They have been employed in the treatment of ailments such as stomach aches, ulcers, and skeletal disorders (Kawarty et al. 2021). Additionally, pine‐derived products have been reported to exhibit therapeutic effects, including appetite stimulation and anti‐diabetic properties (Satil et al. 2011; Güler et al. 2021). In particular studies on the compositional content of pine bark and cones have shown that this antioxidative effect may have a positive functional effect in foods (Hendek Ertop and İncemehmetoğlu 2022). For this purpose, the use of parts of some pine species (*Pinus maritama, Pinus brutia, Pinus nigra, Pinus sylvestris*, etc.) such as bark, needles, and cones as natural food additives has attracted the attention of researchers, but studies to date have been limited (Yesil‐Celiktas et al. 2009; Zhang et al. 2021).

Vinegar, widely used in cuisines, especially in Asian countries (Turkey) is a traditional fermented product widely consumed for its nutritional and medicinal benefits. This product, produced by alcohol and subsequent acetic fermentation of various plants, fruits, grains, and even forest waste such as pine bark, needles, and cones, which are known to have bioactive properties, is common (Ho et al. 2017; Özdemir and Budak 2022; Erturkmen et al. 2024). Although vinegar production is nowadays mostly industrialized, the traditional method of production, in which biological activity is better preserved, is also gaining attention. In vinegar production with the traditional method, the raw material is subjected to slow fermentation that lasts up to several months, and a cloudy gelatinous structure may form due to this long fermentation. In this way, vinegar that does not undergo any heat treatment or filtering process is called live vinegar and preserves all nutrients and biologically active microorganisms (Melkis and Jakubczyk 2024).

Vinegar flavor is affected by alcohol and acetic acid fermentations and various reactions that occur after fermentation. Among those affecting this aroma are organic acids, amino acids, and volatile aroma compounds. In addition, the microbiota of vinegar is critical in this respect. The genes and enzymes of these microorganisms play a role in the formation of flavor substances; they convert different substrates to various flavor compounds and bioactive substances (Jiang et al. 2019; Shi et al. 2022). In vinegar, the final taste depends on the specific ratios of volatile aroma compounds, of which esters, acids, alcohols, ketones, aldehydes, phenols, and oxazoles are the main components and are important primates of acetic acid fermentation in alcohols (Al‐Dalali et al. 2020; Shi et al. 2022).

Anatolian Black Pine vinegar is an emerging product, produced through the fermentation of *Pinus nigra* cones. This vinegar is particularly notable for its complex phytochemical profile, including phenolic compounds, organic acids, and volatile compounds, which contribute to its potential health‐promoting properties. This study aims to comprehensively investigate the functional properties and bioactive profile of traditionally produced pine cone vinegar, with a particular focus on its potential as a natural antimicrobial agent. To achieve this, the physicochemical, sensory, and antimicrobial characteristics of different pine cone vinegar samples were analyzed, alongside their bioactive compounds, including total phenolic content, antioxidant capacity, and organic acid composition. Additionally, their aroma components and cultivable microbiota were examined to gain deeper insights into their microbial ecology. Among the vinegar samples evaluated, CnB pine cone vinegar exhibited distinctive properties, warranting further analysis through metagenomic approaches to elucidate its microbiota. By integrating these findings, this study seeks to enhance the scientific understanding of pine cone vinegar and contribute to expanding its applications in the food and health industries.

## 2 Materials and Methods

### 2.1 Materials

**FIGURE 1:**
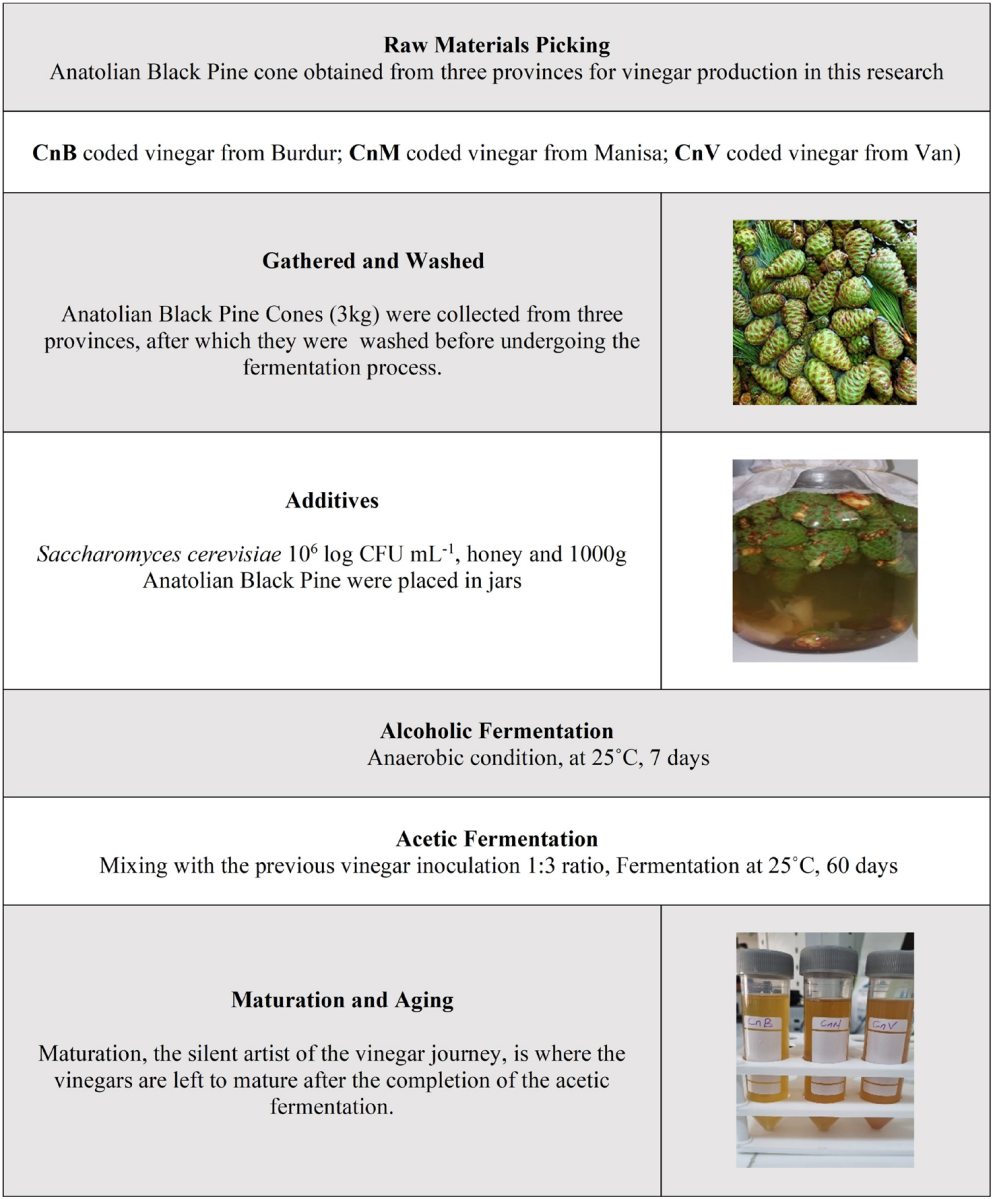
Schematic flowchart depicting the process for production of traditional cone vinegars.

3 kg of Anatolian Black Pine cones were separately obtained from Burdur (CnB), Van (CnV), and Manisa (CnM) provinces for vinegar production in this research. The traditional method was used in vinegar production. Anatolian Black Pine Cone samples were washed and divided into three 1000 g portions, which were placed in 5 L glass jars. Each sample was mixed with 1.5 L of water. Following this, *Saccharomyces cerevisiae* (0.3 g/L) (instant bread yeast, Turkey) and 50 g of honey (Balparmak, Turkey) were employed for ethanol fermentation in the vinegar production process under sealed conditions for 20 days. Subsequently, 150 mL of home‐made apple cider vinegar (Kemal Kükrer, Turkey) was added, and the final volume was adjusted to 5 L with water. The jars were covered with cloth to facilitate oxygen exchange, and acetic acid fermentation proceeded at 28°C–30°C for 60 days, with periodic acidity measurements. Figure 1 shows a schematic diagram illustrating the traditional cone vinegar process.

### 2.2 Physicochemical Parameters

Total soluble dry matter (°Brix) values were measured at 20°C using the Hanna HI 96801 (Hamburg, Germany) refractometer calibrated with distilled water. The pH values of the vinegar samples were measured using a Mettler S220‐K pH meter (Switzerland) (Hannon et al. 2003). Total acidity in % acetic acid was calculated depending on the amount of 0.1 N NaOH solution spent in the titration (Park et al. 2022). HunterLab Colorflex (Management Company, USA) color measurement device was used to determine the color properties of vinegar samples. CIE L*, a*, b* values of the samples were measured after calibrating the device (Kirca et al. 2007).

### 2.3 Total Phenolic Content

The total phenolic content (TPC) of the vinegars was determined using the Folin–Ciocalteu method (Singleton and Rossi 1965; Bulantekin et al. 2023), with the results obtained by a spectrophotometer (Thermo Fisher Scientific, Evolution 300, Massachusetts, USA) at a wavelength of 725 nm. The concentration of TPC was calculated using a calibration curve and reported as milligrams of gallic acid equivalents per liter (mg GAE/L).

### 2.4 Antioxidant Activity

The DPPH radical scavenging activity of vinegar samples was assessed following Cemeroğlu (2013) and Carrasco‐Pancorbo et al. (2007). Vinegar samples (20–100 μL) were mixed with 80% ethanol (to 100 μL) and 3.9 mL of 0.1 mM DPPH solution, stirred, and incubated in the dark for 30 min. Absorbance was measured at 517 nm, and antioxidant capacity was calculated as Trolox equivalents using calibration graphs (*R* ^2^ = 0.99). For TEAC determination, ABTS+ radical cations were prepared by reacting 7 mM ABTS stock solution with 2.45 mM potassium persulfate for 12–16 h in the dark. Antioxidant interaction was quantified by measuring absorbance reduction at 734 nm, with results expressed as TEAC (μmol TE/L) (Re et al. 1999; Bulantekin and Kuşçu 2024).

### 2.5 Determination of Organic Acids

Organic acid analysis was conducted by modifying methods from Alhendawi et al. (1997) and Kordis‐Krapez et al. (2001). Samples (5 mL) were homogenized with 5 mL of 2% H_3_PO_4_, filtered, and diluted with 3 mL of 0.01 M KH_2_PO_4_ (pH 8.0). After filtration through a Supelco C18 cartridge, eluates were analyzed via HPLC using a Teknokroma Tracer Extrasil Ods (2) column (250 × 4.6 mm, 5 μm particle size) with H_3_PO_4_ (pH 2.2) as the mobile phase. The system operated isocratically at a 0.8 mL/min flow rate, 30°C column temperature, and 20 μL injection volume. Detection was performed at 210 nm using an SPD‐10Avp UV–VIS detector.

### 2.6 Determination of Aroma Components

The aroma components of vinegars made from pine cones sourced from Turkey, the provinces of Burdur, Van, and Manisa, were analyzed using gas chromatography/mass spectrometry solid‐phase microextraction (GC/MS‐SPME) (Shimadzu QP 2010 Ultra). The analysis utilized a Restek RTX‐1301 column (60.0 m × 0.25 mm × 1.40 μm) with an injection temperature of 230°C, a pressure of 164.9 kPa, and a column flow rate of 1.50 mL/min. The temperature program started at 40°C (held for 5 min), increased to 230°C, and was held for 20 min. Retention index (RI) values were calculated by comparing peak retention times with those of a hydrocarbon standard.

### 2.7 Sensory Properties

A panel of 20 trained experts, selected per ISO guidelines (38 amendment), evaluated the sensory properties of the products using a 5‐point Likert scale. Familiar with pine cone‐based food products (e.g., cone molasses), the panel assessed appearance, color, texture, taste, odor, and overall acceptance. The evaluation took place in a sensory lab meeting ISO standards (41 amendment), under standard lighting at 21°C. Panelists were advised to consume water between samples to minimize residue effects.

### 2.8 Culturable Viable Microorganism Compositions

To determine the number of viable microorganisms present in vinegars produced from pine cones sourced from different provinces, vinegar samples were collected, and serial dilutions were prepared. The enumeration of viable microorganisms was performed through serial plating on seven different selective growth media to assess the culturable microbial diversity in pine cone vinegar. Total aerobic mesophilic bacteria (TAMB) were enumerated using Plate Count Agar (PCA) after incubation at 30°C for 48 h. Acetic acid bacteria were counted on Glucose‐Yeast Extract‐Calcium Carbonate (GYC) medium (50 g/L glucose, 10 g/L yeast extract, 20 g/L agar, 3 g/L CaCO_3_, and 0.6% ethanol‐acetic acid mixture, pH 6.8) supplemented with 100 mg/L pimaricin (Sigma‐Aldrich, Steinheim, Germany) to inhibit yeast and mold growth. The plates were incubated aerobically at 30°C for 3–4 days, and distinct colonies on GYC medium were selected for the count of dominant bacterial species. Lactic acid bacteria were enumerated using De Man, Rogosa, and Sharpe (MRS) agar (Merck, Germany) at 30°C for 48 h. Yeasts and molds were cultivated on Potato Dextrose Agar (PDA) with lactic acid (pH 3.5), Yeast Extract Glucose Chloramphenicol (YGC) agar, and Rose Bengal Chloramphenicol Agar (RBCA), with incubation at 25°C for 3–5 days (De Vero et al. 2006; Maragkoudakis et al. 2006).

### 2.9 Antibacterial Activity

The agar well diffusion method was used to screen the antimicrobial activity. The reference cultures used in the study were *Escherichia coli* (ATCC‐25922), *Staphylococcus aureus* (ATCC‐6538) and *Enterococcus faecalis* (ATCC‐19433) obtained from culture collections of Ardahan University. All the bacterial strains were overnight incubated at 37°C after their inoculation into Tryptic Soy Broth (TSB‐Merck). The optical density of each culture was adjusted to 0.5 McFarland standard (10^8^ bacterial cells/mL) in Phosphate‐Buffered Saline (PBS). They were transferred to Petri dishes containing Mueller Hinton Agar (MHA‐Merck) and 100 μL inoculum was spread on MHA. A sterilized 6 to 8 mm cork borer was used to make agar wells; 25 μL, 50 μL, and 100 μL of the vinegar were placed into each well. The zones formed around the wells were measured in mm with a ruler (Bioanalyse) and activity was determined (Özturk et al. 2015).

### 2.10 The Metagenomic Analysis of CnB

The microbial composition of CnB, including the 16S V3‐V4 and ITS2 gene region structures, was analyzed using metagenomic sequencing at the BM Labosis laboratory (Ankara, Turkey). The samples were centrifuged at 10.000 × g for 10 min, then separated and immediately used for DNA extraction. Genomic DNA was extracted from the samples using GeneMATRIX Tissue & Bacterial DNA (EurX, Poland) and GeneMATRIX Plant & Fungi DNA (EURx, E3595, Poland) isolation kits, following the manufacturer's instructions. The integrity and concentration of the extracted DNA were assessed using spectrophotometry (OD260/280), fluorometry (Qubit 2.0 Fluorometer), and 1% agarose gel electrophoresis. DNA samples with concentrations above 1 μg were used for library construction. Total eDNA was used as a template and the 16S V3‐V4 and ITS2 gene region was selected for metagenomic analysis. After performing the Poly(A) tailing, the samples were ligated with a full‐length adapter for Illumina sequencing and subsequently subjected to PCR amplification. The PCR products were purified using the AMPure XP (Beckman Coulter) system and quantified by real‐time PCR. The sequencing libraries were generated, and index codes were added using the Nextera XT index kit (FC‐131‐1001/FC‐131‐1002) for Illumina according to the manufacturer's guidelines. QIIME 2 software was used for the determination of taxonomic species (Bolyen et al. 2018).

### 2.11 Statistical Analysis

Statistical software Minitab 17 (USA) was used for the mean ± standard deviation results between properties such as physicochemical properties and antimicrobial activity, and these analyses were performed in three replicates. The statistical evaluation of the sensory analysis results was performed using SPSS 26.0 (SPSS Inc., Chicago, USA). The Kruskal–Wallis test, a nonparametric test, was used to determine the differences among the means of the groups in evaluating the sensory analysis results of vinegar.

## 3 Results

### 3.1 Physicochemical Parameters of Traditional Cone Vinegars

Dry matter, °Brix, pH, and total acidity, L*, a*, and b* values of vinegar samples are given in Table 1. While the values of CnB (3.94) and CnV (3.90) were very close in total dry matter of vinegars, this value was lower in CnM (2.97). The highest°Brix value was obtained in vinegar coded CnB (4.87), followed by CnV (3.85) and CnM (3.12). The pH and total acidity values of CnB, CnV, and CnM samples were determined in the range of 3.23–3.37 and 15.14–17.09 g/L, respectively. Also, the L*, a*, and b* values in vinegar samples were determined in the ranges of 11.61–13.64, 2.30–2.54, and 11.68–13.18, respectively.

**TABLE 1:** Physicochemical and bioactive properties of all cone vinegar.

Parameters	CnB	CnV	CnM
Total dry matter (%)	3.94 ± 0.02^a^	3.90 ± 0.03^a^	2.97 ± 0.04^b^
°Brix (%)	4.87 ± 0.04^a^	3.85 ± 0.09^b^	3.12 ± 0.04^c^
pH	3.23 ± 0.01^c^	3.30 ± 0.02^a^	3.37 ± 0.02^b^
Total acidity (g/L)	17.09 ± 0.42^a^	15.41 ± 0.54^b^	15.14 ± 0.50^b^
L*	12.62 ± 0.01^b^	13.64 ± 0.02^a^	11.61 ± 0.01^c^
a*	2.30 ± 0.28^a^	2.54 ± 0.05^a^	2.42 ± 0.11^a^
b*	12.38 ± 0.30^b^	13.18 ± 0.50^a^	11.68 ± 0.02^b^
Acetic acid (μg/mL)	283046.42 ± 1521.63^a^	264587.96 ± 1985.63^c^	271463.68 ± 1763.22^b^
Ascorbic acid (μg/mL)	520.93 ± 24.45^a^	480.63 ± 17.56^c^	491.45 ± 26.87^b^
Oxalic acid (μg/mL)	175.89 ± 9.63^a^	162.86 ± 12.58^c^	164.86 ± 13.62^b^
Fumaric acid (μg/mL)	25.12 ± 1.96^a^	17.74 ± 0.96^c^	19.45 ± 1.22^b^
TPC (mg GAE/L)	163.88 ± 5.23^c^	167.92 ± 4.24^b^	174.179 ± 3.87^a^
DPPH (μmol TE/L)	1050.92 ± 50.54^c^	1083.14 ± 63.65^b^	1090.23 ± 54.36^a^
ABTS (μmol TE/L)	1170.75 ± 81.28^c^	1176.45 ± 74.49^b^	1179.25 ± 64.53^a^

*Note:* The experiments were conducted in triplicate, and the acquired data were reported as mean ± standard deviation. The letters 'a, b, c' indicate significant differences (*p* < 0.05).

### 3.2 Bioactive Properties of Cone Vinegar Samples

The concentrations of acetic, ascorbic, oxalic, and fumaric acids in all tested vinegars were measured within the ranges of 264587.96–283046.42 μg/mL, 480.63–520.93 μg/mL, 162.86–175.89 μg/mL, and 17.74–25.12 μg/mL, presented in Table 1, respectively. The total phenolic content (TPC) was determined as 163.88 mg GAE/L for CnB, 167.92 mg GAE/L for CnV, and 174.79 mg GAE/L for CnM, indicating slight variations among the samples. The antioxidant activity, assessed through DPPH and ABTS assays, also exhibited differences among the vinegars. DPPH was measured as 1050.92 μmol TE/L for CnB, 1083.14 μmol TE/L for CnV, and 1090.23 μmol TE/L for CnM. Similarly, ABTS was recorded as 1170.75 μmol TE/L for CnB, 1176.45 μmol TE/L for CnV, and 1179.25 μmol TE/L for CnM.

### 3.3 Culturable Viable Microorganism Composition

The results of viable microorganism count analyses for traditional cone vinegar samples are presented in Table 2. Microbiological analyses revealed total aerobic mesophilic bacteria (TAMB) counts ranging from 3.89 to 5.56 log CFU/mL, *Lactobacillaceae* counts between 2.94 and 4.55 log CFU/mL, acetic acid bacteria counts from 6.15 to 7.36 log CFU/mL, and yeast‐mold counts varying between 2.43 and 6.91 log CFU/mL. Acetic acid bacteria values were determined in CnV and CnM vinegar at averages of 6.90 log CFU/mL^−1^ and 6.15 log CFU/mL^−1^, respectively. The total viable count of acetic acid bacteria, known as the dominant species of vinegar, determined in CnB vinegar an average of 7.36 log CFU mL‐1 as well as logarithm higher than other pine cone vinegar. When the results were examined, it was determined that the aerobic plate count value of the CnB vinegar samples was 4.90 log CFU/mL^−1^, and the *Lactiplantibacillus* was 4.55 log CFU/mL^−1^. The count of yeasts and mold was determined in the 3.45–5.81 log CFU/mL^−1^ range in CnB with different media.

**TABLE 2:** Enumeration of culturable microbiota with seven different media for the bacteria and yeast.

Media	CnB	CnV	CnM
MRS	4.55 ± 0.07^a^	3.45 ± 0.38^b^	2.94 ± 0.04^b^
PCA	4.90 ± 0.06^b^	3.89 ± 0.07^c^	5.56 ± 0.31^a^
GYC	7.36 ± 0.15^a^	6.90 ± 0.10^b^	6.15 ± 0.27^c^
PDA	5.81 ± 0.21^b^	5.45 ± 0.38^b^	6.91 ± 0.04^a^
YGC	3.45 ± 0.12^ab^	2.43 ± 0.35^b^	4.14 ± 0.77^a^
RBCA	3.39 ± 0.30^a^	3.12 ± 0.07^a^	3.82 ± 0.53^a^

*Note:* The experiments were conducted in triplicate, and the acquired data were reported as mean ± standard deviation.

Abbreviations: GYC, glucose yeast extract calcium carbonate agar (50 g/L glucose, 10 g/L yeast extract, 20 g/L agar and 3 g/L CaC0_3_ and 0.6% 1:1 ethanol and acetic acid); MRS, De Man rogosa and sharpe; PCA, plate count agar; PDA, potato dextrose agar (pH 3.5); RBCA, rose bengal chloramphenicol agar; YGC, yeast extract glucose chloramphenicol agar. The letters 'a, b, c' indicate significant differences (p < 0.05).

### 3.4 Antibacterial Activity of Vinegars

In the present study, the antimicrobial effects of vinegar at different rates against 3 different pathogens ( *Escherichia coli* [ATCC‐25922], *Staphylococcus aureus* [ATCC‐6538], *Enterococcus faecalis* [ATCC‐19433]) are presented in Table 3. In the study, the antimicrobial effects of 3 different amounts, 25 μL, 50 μL and 100 μL, on microorganisms were investigated. While all three doses created zones in this vinegar, the trial results, especially with 100 μL, are quite effective. Smaller zones were obtained from all tested amounts in the vinegar sample coded CnV compared to CnB. When the results are examined, it is seen that the effect of 25 μL is generally lower. When all results are considered, the CnM sample was determined to be the weakest in terms of antimicrobial results. While no zone was formed at the 25 μL dose, the effectiveness of the 100 μL results was quite low compared to the other two vinegars.

**TABLE 3:** Antibacterial activity of all traditional vinegars.

Vinegar	*Doses*	Pathogens		
		*S. aureus*	*E. coli*	*E. feacalis*
CnB	25 mL	22.66 ± 0.57^c^	15.83 ± 0.28^c^	17.06 ± 0.11^c^
	50 mL	30.16 ± 0.28^b^	32.33 ± 0.57^b^	20.33 ± 0.57^b^
	100 mL	33.67 ± 0.56^a^	44.91 ± 0.17^a^	24.94 ± 0.11^a^
CnV	25 mL	10.93 ± 0.11^c^	11.84 ± 0.28^c^	8.03 ± 0.05^c^
	50 mL	14.91 ± 0.17^b^	21.01 ± 0.57^b^	15.81 ± 0.34^b^
	100 mL	26.91 ± 0.17^a^	22.95 ± 0.05^a^	21.34 ± 0.55^a^
CnM	25 mL	ND*	ND*	ND*
	50 mL	8.94 ± 0.11^b^	11.01 ± 0.11^b^	8.97 ± 0.05^b^
	100 mL	10.71 ± 0.51^a^	15.06 ± 0.11^a^	11.06 ± 0.11^a^

*Note:* The mean ± standard deviation results of three replicates are presented.

* In the same row indicates the results of the vinegar doses against pathogens with significant differences (*p* < 0.05). The letters 'a, b, c' indicate significant differences (*p* < 0.05), ND* Not detected.

### 3.5 Aroma Components of Cone Vinegar Samples

The volatile compounds in the vinegars tested in the current study are presented in Table 4. The total scores of these volatile compounds in CnB, CnV, and CnM were determined as follows: alcohols (0.84%, 0.67%, and 0.58%), esters (13.31%, 13.17%, and 13.74%), acids (74.49%, 74.07%, and 73.76%), terpenes (6.51%, 6.80%, and 6.85%), ketones (4.24%, 4.38%, and 4.44%), phenols (0.43%, 0.51%, and 0.45%) and other compounds (0.11%, 0.26%, and 0.18%), respectively. Among these, ethyl acetate, acetic acid, isobutyl acetate, 2‐methylbutyl acetate, 4‐terpineol, alpha‐terpineol, verbenone, and 2‐phenethyl acetate draw attention.

**TABLE 4:** Aroma volatiles in all tested vinegars (% of total area).

Group	Compound	CnB (%)	CnV (%)	CnM (%)
Acids	Acetic acid	74.14 ± 0.18	73.59 ± 0.28	73.35 ± 0.32
	Isovaleric acid	0.35 ± 0.05	0.48 ± 0.09	0.41 ± 0.08
	Total acids	74.49	74.07	73.76
Esters	Ethyl acetate	8.60 ± 0.78	8.40 ± 0.48	8.45 ± 0.66
	2‐Methylbutyl acetate	1.63 ± 0.12	1.51 ± 0.22	1.72 ± 0.14
	2‐Phenethyl acetate	1.48 ± 0.23	1.50 ± 0.19	1.63 ± 0.27
	D,L‐2,3‐Butanediol diacetate	0.14 ± 0.03	0.20 ± 0.06	0.21 ± 0.08
	Isoamyl acetate	0.70 ± 0.12	0.59 ± 0.14	0.82 ± 0.11
	Isobutyl acetate	0.49 ± 0.11	0.47 ± 0.17	0.45 ± 0.09
	Methyl acetate	0.22 ± 0.05	0.31 ± 0.11	0.28 ± 0.08
	Myrtenyl acetate	0.08 ± 0.02	0.19 ± 0.08	0.13 ± 0.03
	Isobornyl acetate	ND*	ND*	0.05 ± 0.01
	Total esters	13.31	13.17	13.74
Terpenes	Alpha‐ Terpineol	3.13 ± 0.81	3.36 ± 0.49	3.01 ± 0.78
	Verbenone	1.95 ± 0.36	1.89 ± 0.12	2.14 ± 0.55
	Pinocamphone	0.70 ± 0.12	0.72 ± 0.14	0.97 ± 0.17
	trans‐Pinocarveol	0.37 ± 0.10	0.62 ± 0.12	0.45 ± 0.10
	Borneol	0.27 ± 0.06	0.21 ± 0.09	0.20 ± 0.06
	4‐Terpineol	0.09 ± 0.04	ND*	0.08 ± 0.02
	Total terpenes	6.51	6.80	6.85
Ketones	Acetoin	3.86 ± 0.56	3.73 ± 1.04	3.91 ± 0.88
	2,4,5‐Trimethyl‐1,3‐dioxolane	0.24 ± 0.07	0.37 ± 0.12	0.31 ± 0.11
	3‐Methyl‐2‐butanone	0.18 ± 0.04	0.28 ± 0.05	0.22 ± 0.08
	Total ketones	4.24	4.38	4.44
Alcohols	Ethyl alcohol	0.60 ± 0.12	0.53 ± 0.13	0.51 ± 0.08
	2‐Methyl‐1‐butanol	0.12 ± 0.03	0.06 ± 0.01	ND*
	3‐Methyl‐1‐butanol	0.04 ± 0.01	0.08 ± 0.03	ND*
	Phenethyl alcohol	0.08 ± 0.03	ND*	0.07 ± 0.02
	Total alcohols	0.84	0.67	0.58
Phenols	2,4‐Di‐tert‐butylphenol	0.32 ± 0.09	0.25 ± 0.06	0.38 ± 0.10
	2,6‐Di‐tert‐Butylquinon	0.11 ± 0.04	0.26 ± 0.09	0.07 ± 0.03
	Total phenols	0.43	0.51	0.45
Other	2,5‐Dimethylfuran	0.11 ± 0.03	0.26 ± 0.07	0.18 ± 0.05
	Total furans	0.11	0.26	0.18

*Note:* ND* Not detected.

### 3.6 Sensory Properties of Traditional Cone Vinegar

**FIGURE 2:**
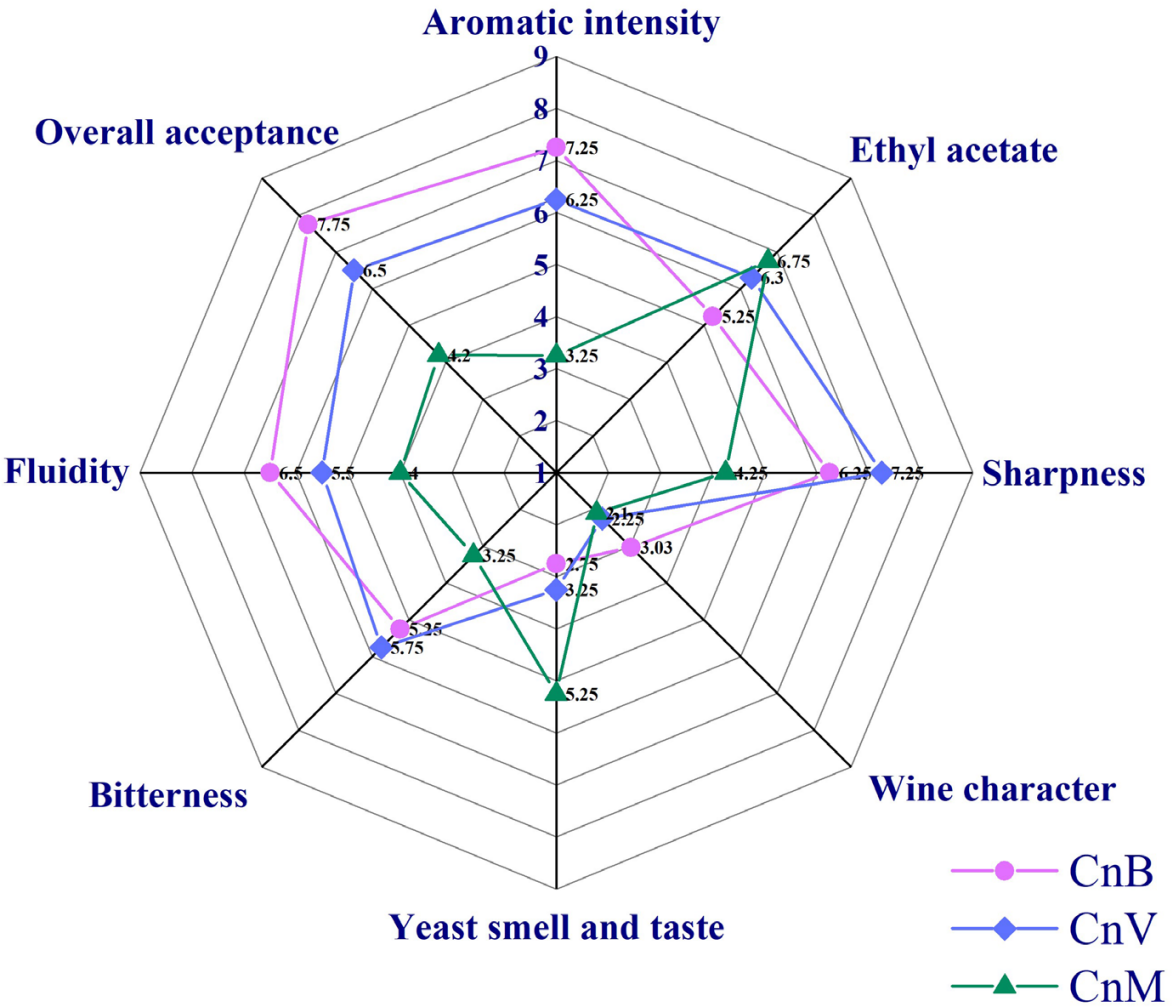
Sensory analysis scores of all tested vinegar.

The results of sensory properties of all cone vinegar samples are presented in Figure 2. The aromatic intensity score value of the samples ranged from 5 to 8. The bitterness results were determined as 5.25, 5.75, and 3.25 CnB, CnV, and CnM, respectively. In the present study, the evaluation scores for ethyl acetate are 5.25. This result is attributed to the high Acetobacteraceae count. The sharpness score of CnB‐coded vinegar was determined as 6.25. This value was determined as 7.25 for CnV and 3.25 for CnM. Sharpness values were consistent with total acidity values. The evaluation scores for wine characters are 3.03, 2.25, and 2.0 for CnB, CnV, and CnM, respectively. This result is attributed to low phenyl ethyl alcohol production, especially for CnB vinegar. When the yeast aroma and taste of the vinegar samples were evaluated, a low score of 2.75 was obtained in CnB, and CnV is followed with a 3.25 score. The worst result was determined in CnN with 5.25; unfortunately, this odor is mostly prominent in vinegar. In the study, isolaleric and fumaric acid production was found in CnB vinegar.

### 3.7 Taxonomic Diversity of CnB

**FIGURE 3:**
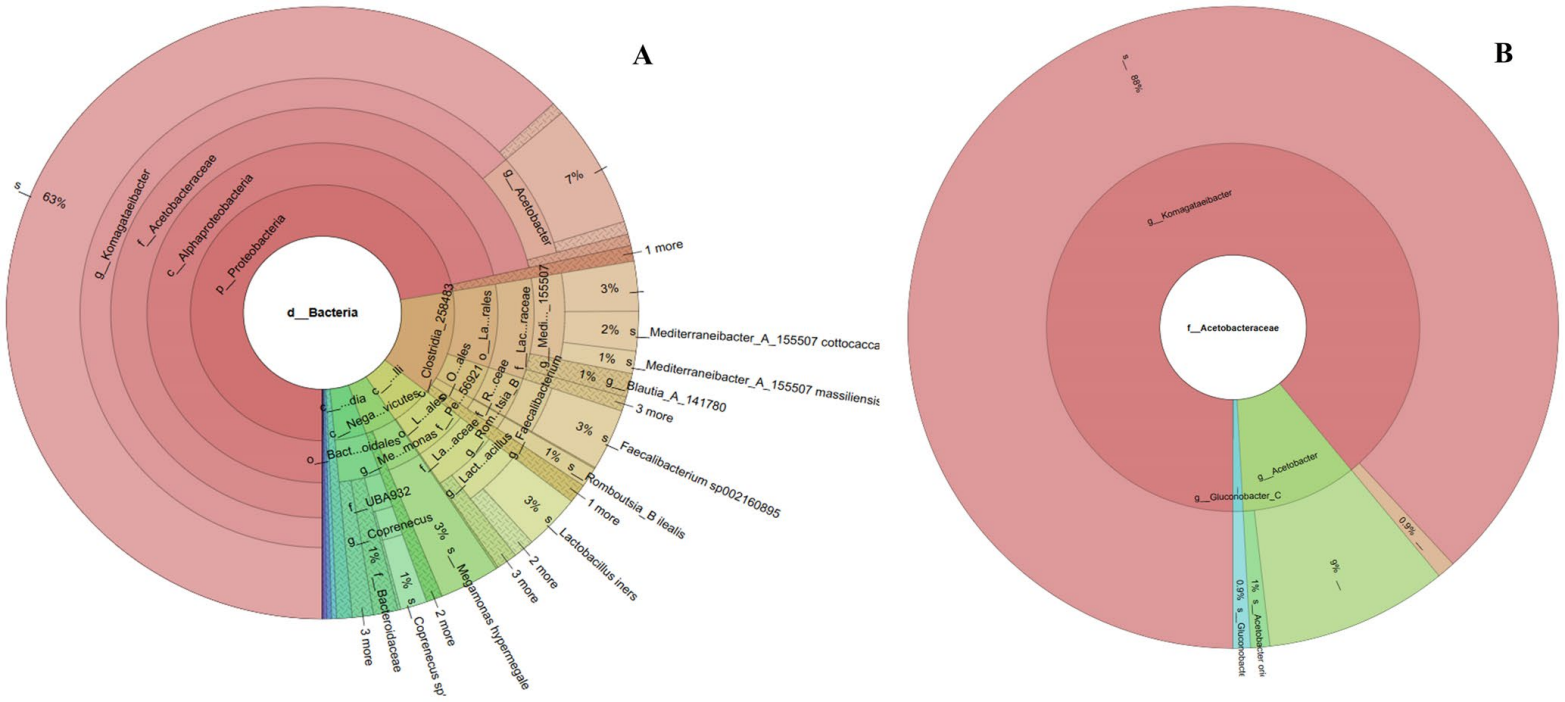
Total bacterial (A) and acetobacter (B) composition of microbial communities present in CnB vinegar. *Gene Region primer sequence (F → R): Universal specific reverse and forward primer set for bacteria 16S V3‐V4; 31 F: TCGTCGGCAGCGTCAGATGTGTATAAGAGACAGCCTACGGGNGGCWGCAG; 805 R:GTCTCGTGGGCTCGGAGATGTGTATAAGAGACAGGACTACHVGGGTATCTAATCC.

**FIGURE 4:**
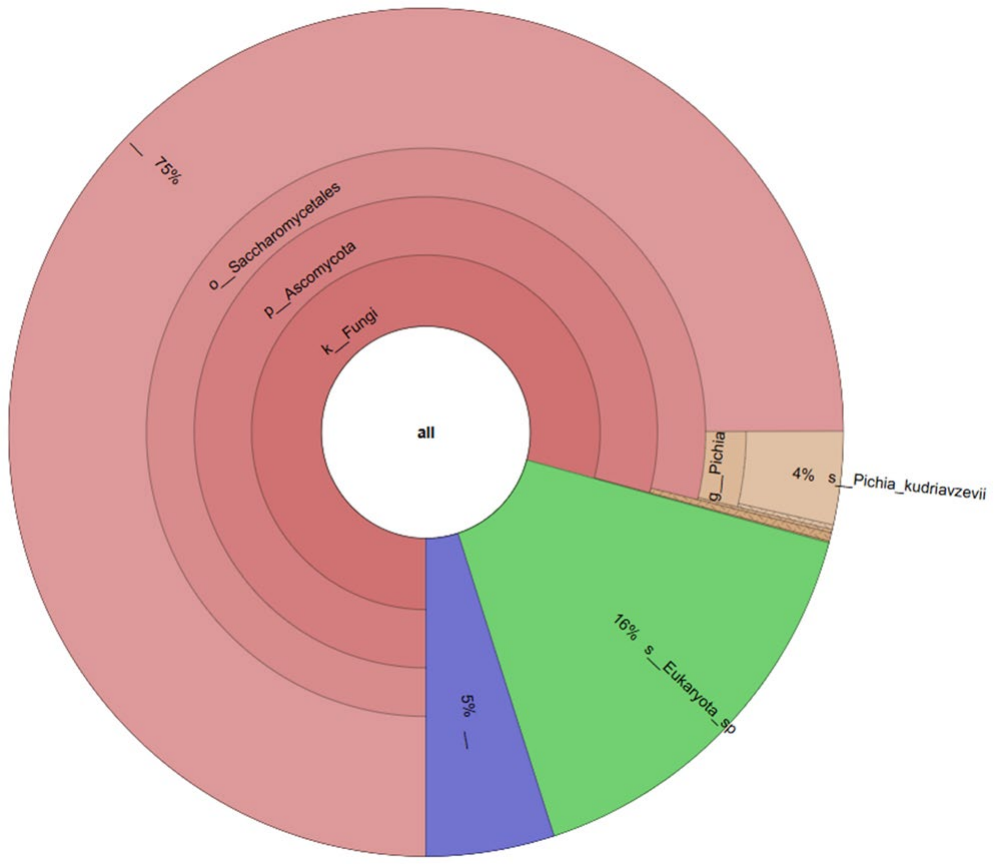
Fungal composition of microbial communities present in CnB vinegar. *Gene Region primer sequence (F → R): Universal specific reverse and forward primer set for mold‐yeast ITS3: TCGTCGGCAGCGTCAGATGTGTATAAGAGACAG GCATCGATGAAGAACGCAGC; ITS4: GTCTCGTGGGCTCGGAGATGTGTATAAGAGACAGTCCTCCGCTTATTGATATGC.

Figure 3 and Figure 4 display a Crona visualization of the microbial communities in CnB vinegar. Six bacterial phyla were identified in this study: *Proteobacteria* (72.296%), *Firmicutes* (22.062%), *Bacteroidota* (3.665%), *Actinobacteriota* (0.791%), *Desulfobacterota* (0.252%), and *Verrucomicrobiota* (0.252%). At the end of fermentation, the dominant bacterial communities at the phylum level were *Acetobacteraceae* (71.47%), *Clostridia* (13.187%), *Bacilli* (5.066%), *Bacteroidetes* (3.665%), and *C. negativicutes* (3.737%). Two fungal phyla, *Ascomycota* (78.717%) and *Eukaryota Incertae sedis* (15.840%), with an abundance of ≥ 0.01%, were present during CnB vinegar fermentation. Additionally, 4.963% of fungal phyla were undetected. Taxonomic analysis at the genus level identified 22 genera with an abundance ≥ 0.01%.

## 4 Discussion

The variations observed in the Brix values of the pine cone vinegar samples were attributed to several factors, including the composition of the raw materials used in production, the efficiency of the fermentation process, the formation of various components during fermentation, and the concentration of dry matter dissolved in water. The dry matter and°Brix values determined in this study were comparable to those reported for blueberry and mulberry vinegars in the studies by Tomar et al. (2020) and Şengün and Kılıç (2018). The reported range of Brix values in fruit vinegars is between 1.20 and 6.63 (Öztürk 2022), with the values found in this study falling within this range. Total acidity and pH values determined in the study were found to be compatible with various vinegar varieties such as banana peel, mango, rice, and apple (Elijah and Etukudo 2016; Adebayo‐Oyetoro et al. 2015; Bayram et al. 2018). Color, a key attribute in how consumers evaluate food, plays a critical role in detecting quality issues and potential defects (Özbek 2023). The color of the raw materials significantly influences the color of vinegar, the production stages, and the methods employed during its manufacture (Ubeda et al. 2011; Jo et al. 2015). The relatively low L* value observed in traditional cone vinegar can be attributed to the absence of high‐temperature treatments such as pasteurization, which helps preserve its natural color characteristics.

Yeasts are a key group of microorganisms involved in vinegar production, contributing to fermentation processes, whereas molds are considered undesirable due to their spoilage activity (Şengün et al. 2022). CnM vinegar, produced using pine cones from Manisa province, exhibited notable findings in its culturable microbiological flora. Yeast and mold counts ranged from 3.82 to 6.92 log CFU/mL, the highest among all vinegar samples. This elevated cell count was linked to sensory analysis results, where panelists reported a distinct yeast and mold odor in this sample. In contrast, the population of lactic acid bacteria (LAB) in CnM was lower compared to the other vinegars, and overall living cell counts across all media indicated a weaker microbiota for this vinegar. On the other hand, CnB vinegar, made with pine cones from Burdur province, was identified as the richest sample in terms of culturable microbiota, highlighting significant variability in microbial diversity and composition between the vinegars. The distinctiveness of flora can have an effect on the varied properties of vinegar.

Acidic fermented products like vinegar can inhibit the growth or survival of pathogenic microorganisms, primarily due to their acidity levels. Weak acids, such as acetic acid, exert antimicrobial effects through various mechanisms, the most common being disruption of cell wall structure and intracellular ATP depletion (Karapinar and Gönül 1992; Özturk et al. 2015; Gökırmaklı et al. 2019). The antimicrobial efficacy of acetic acid is influenced by factors such as its acid ionization constant (Ka), environmental pH and temperature, the presence of other antimicrobial agents, and the specific type of target pathogen (Ayhan and Bilici 2015). In this study, pathogen sensitivity to vinegar varied among samples, with the most pronounced antimicrobial effects observed in the CnB vinegar. Notably, CnB exhibited strong activity against *E. coli* and *S. aureus* at both 25 mL and 50 mL doses. This result contrasts with the findings of Özturk et al. (2015), where vinegar samples at a dose of 50 μL showed no inhibition zones against these pathogens. The superior antimicrobial performance of the CnB sample underscores its potential as an effective natural antimicrobial agent.

Among the organic acids found in vinegar, lactic acid and acetic acid are the main organic acids and constitute more than 90% of the total acidity (Chai et al. 2020). *Streptomycetaceae* and *Firmicutes*, especially *Acetobacter* and *Bacillus* bacteria, which are also generally found in the vinegar microbiota, convert pyruvate into acetic acid using pyruvate oxidase and pyruvate dehydrogenase enzymes (Jiang et al. 2019; Shi et al. 2022). During the alcohol fermentation and acetic acid fermentation stages, the acetic acid content continues to increase and provides the basic flavor (Jiang et al. 2019). The formation of organic acids, especially the high acetic and ascorbic acid content obtained in the present study, was mainly attributed to the metabolic functions of the microbiota that secrete these functional enzymes. The ascorbic acid determined in cone vinegars was determined to be much higher than the results found in date, pomegranate, apple, grape, hawthorn, rosehip fruit, and rose produced in previous studies (Özdemir and Budak 2022; Özdemir et al. 2022; Özdemir, Pashazadeh et al. 2022). Additionally, researchers stated that the high amounts of acetic and ascorbic acidcontribute to the increase of antioxidant activity (Neffe‐Skocińska et al. 2023).

The antioxidant activity of fruits and vegetables and their products is affected not only by the content of polyphenols but also by various factors such as the type of raw material, the production method, and vitamin content (Jo et al. 2012). The phenolic content in CnB vinegar was found to be lower compared to the other vinegars. However, its ascorbic acid concentration was notably higher, which may contribute significantly to enhancing the antioxidant capacity of pine cone vinegar despite its lower phenolic content. The TPC values observed in pine cone vinegars in the present study are comparable to those reported in Ubeda et al. (2011) and Ubeda et al. (2013) studies. Researchers reported that total phenolic content values for persimmon vinegar ranged from 268 to 397.5 mg GAE/L and for strawberry vinegar ranged from 683 to 781 mg GAE/L. Similarly, Kahraman et al. (2022) determined TPC levels in apple and grape vinegars as 209.10 mg GAE/L and 498.36 mg GAE/L, respectively. DPPH values of cone vinegar in this study were found to be higher than the DPPH values of some apple (Kadiroğlu 2018), grape, and blueberry vinegar (Tomar et al. 2020) in previous studies and were found to be lower than strawberry (Ubeda et al. 2013) and grape vinegar (Kadiroğlu 2018). These findings underscore the substantial variability in phenolic compound content among different types of vinegar, highlighting the critical role of raw materials in shaping the polyphenolic profile and, consequently, the functional properties of vinegar.

One of the important parameters for the consumer in vinegar is its aroma properties. The current study determined that various organic acids in the vinegar include acetic acid, oxalic acid, ascorbic acid, and formic acid. Among these, acetic acid was identified as the dominant compound, playing a pivotal role in shaping the characteristic taste profile of the vinegar. Studies about vinegar have shown that the aroma and overall acceptability scores of vinegar mostly correlate with short‐chain volatile organic acids (Sossou et al. 2009). At the end of the fermentation process, a total of 27 aroma compounds were identified in the pine cone vinegars, including nine esters, six terpenes, four alcohols, two acids, three ketones, two volatile phenols, and one other compound. Gamli (2022) identified α‐terpineol, terpinene‐4‐ol, endo‐borneol, α‐curcumene, and sorbic acid as the primary aroma components in pine cones. Similarly, Ucar and Balaban (2004) analyzed the volatile compounds in Anatolian Black Pine ( *Pinus nigra* ) species and highlighted their richness in components such as pinocarveol, borneol, terpineol‐4, and α‐terpineol, several of which were also detected in the present study. These compounds significantly contribute to the organoleptic characteristics of cone vinegar, resulting in a product with both aromatic and bioactive properties. In this study, an average of 8.48% ethyl acetate and 1.54% 2‐Phenethyl acetate of ester compounds, which facilitates the formation of various sweeteners and gives various aromas such as flower, fruit, honey, and rose (Jeong et al. 2011; Gao et al. 2017; Khaleel et al. 2018; Sales et al. 2020), was detected in all vinegars produced. Borneol, a significant bicyclic monoterpene in terms of its medical and functional properties (Hong et al. 2024) was identified in all three vinegars examined in this study. The highest concentration was detected in CnB, at 0.27%, followed by CnV (0.21%) and CnM (0.20%), respectively. This compound contributes significantly to the overall value of vinegars, particularly with respect to their functional properties. In the present study, the terpene content of the vinegars was determined as 6.72% on average. Studies have reported that terpineols, which are monocyclic monoterpenoid tertiary alcohols, have various properties such as antioxidant, anti‐inflammatory, anticonvulsant, antimicrobial, anticarcinogenic, etc. (Sales et al. 2020). Karklina and Ozola (2023) stated that these compounds can also vary depending on the place of cultivation, tree variety, harvest time, and storage conditions. But the present study detected similar amounts of α‐terpineol in all vinegars. Another important terpineol is 4‐Terpineol (4‐TA), a monocyclic monoterpene aroma compound and the primary component of tea tree essential oil, known for its antibacterial and antioxidant activities due to its ring structure (Cheng et al. 2024). In the current work, 4‐TA was not detected in CnV vinegar, whereas its concentration was determined to be 0.08% in CnM and 0.09% in CnB. The antibacterial properties of 4‐TA may have a positive contribution to the good well diffusion results of CnB and CnM vinegars. This phenomenon underscores the functional properties of pine cone vinegar, thus prompting further investigation into its potential applications. In the present study, different amounts of compounds belonging to the phenol group, 2,4‐di‐tert‐butylphenol and 2,6‐di‐tert‐butylquinone, and in the ketone group, Acetoin, 2,4,5‐trimethyl‐1,3‐dioxolane and 3‐methyl‐2‐butanone were detected in all vinegars. In the formation of these compounds, it is stated that the metabolic activities of especially acetic acid bacteria are effective (Özdemir, Pashazadeh et al. 2022).

In the current study, isobutyl acetate, which is produced by *Acetobacter* and *Lactobacillaceae species* and is stated to give a fruity taste to vinegar (Fang et al. 2021), was detected in vinegars, and the highest amount was determined in CnB with 0.49%. Acids such as isovaleric acid and fumaric acid, which are known to give vinegar a stale taste, are produced by lactic acid bacteria (Zhang et al. 2017; Fang et al. 2021). In the sensory evaluation, the highest score for the sharpness feature, which is a feature related to acidity, was also obtained in this vinegar. These findings indicate a positive correlation between the results of the aroma analysis and those of the sensory evaluation, thereby corroborating each other. 3‐Methyl‐1‐butanol and ethanol are mostly produced by *Saccharomyces*, *Pichia*, and *Lactococcus*, and they give a predominantly malt taste (Fang et al. 2021). In this study, it was detected only in vinegar CnB (0.04%).

Sourness is the main sensory characteristic of vinegar; organic acids are effective in giving this taste to vinegar. In this study, ethyl acetate evaluation scores were notably high, with CnB vinegar achieving a score of 5.25, indicating strong intensity. In contrast, CnV vinegar was perceived as harder to drink due to its sharpness, whereas CnM lacked the characteristic sharp taste typically associated with vinegar. During vinegar production, alcohol residues may remain due to the high formation of acetic acid, which can give vinegar a wine‐like character (Góamez et al. 2006). When the yeast aroma and taste of the vinegar samples were evaluated, a low score of 2.75 was obtained in CnB, and CnV was followed by a score of 3.25 The highest score for the aromatic intensity attribute was observed in CnB, with a value of 7.25 among the vinegars in the study. This was followed by CnV, with a score of 6.25, and CnM, with a score of 3.25. In terms of aromatic intensity, CnM vinegar was found to exhibit a relatively weak profile in comparison to the other two vinegars. The yeast smell and taste are also regarded by consumers as important and unpleasant criteria. In this regard, CnM was again the most disliked vinegar, with the highest score (5.25). This was attributed to its high yeast count, which is perceived as a negative quality by consumers. In terms of general acceptability, CnB (7.25) was the most favorite vinegar among the three vinegars used in the study. It was followed by CnV (6.50) and CnM (4.2). It was observed that the aromatic components and microbial effect of the vinegars were quite high in general acceptance.

The results of the analyses indicated that the CnB vinegar, produced using cone from Burdur province, came to the forefront. The vinegar displayed notable bioactive components and culturable microorganisms. Metagenomic sequencing can help calculate relative abundances at different levels of microbial communities to reveal diversity and variations in microbial populations (Gao et al. 2024). In the current work, metagenomic analysis was carried out to determine in more detail the microbial composition of CnB vinegar, which has come to the forefront of antimicrobial properties and culturable bacterial diversity. Fungal genera (i.e., *Saccharomyces*, *Rhizosphaera*, and *Pichia*) were dominant (> 1% abundance) during CnB vinegar fermentation; on the whole microbiota profiles, the diversity and relative abundance of bacteria were richer than that in fungi both at phylum and genus levels during CnB vinegar fermentation. In most types of vinegar fermentation, members of phyla *Firmicutes*, *Proteobacteria*, and *Ascomycota* are the dominant microorganisms (Gao et al. 2024). This succession tendency may have resulted from the high acidity. *Acetobacter*, *Clostridia*, and *Bacilli* are the main microorganisms that can survive in high acidity conditions, and *Bacteroides*, which are anaerobes with high abundance during fermentation, are inhibited by high acidity. These results were consistent with previous findings on different kinds of vinegar fermentation (Peng et al. 2015; Wu et al. 2021). For the fungal phylum, the relative abundance of *Saccharomyces* was 78.717% at the end of the fermentation. *Saccharomyces* had the highest relative abundance among the fungal genera and played an important role as the main producer of ethanol. They can rapidly consume low‐molecular‐weight sugar, thereby increasing the alcohol degree (Gong et al. 2021). A high alcoholic condition could inhibit the growth and metabolism of most fungal communities (Li et al. 2016). Therefore, members of the genus *Saccharomyces* possess an absolute advantage during fermentation. Even though only a few molds were involved in the fermentation, they played a main role in the final quality at the end of the fermentation of vinegar because they could produce a wide range of secondary metabolites and many kinds of enzymes (Erturkmen et al. 2024). This aroma richness and sensory preferability determined in pine cone vinegar is because the bacterial and yeast‐mold microbiota can be attributed to the richness of enzymes it has.

## 5 Conclusions

Pine cone vinegar, a product derived from the fermentation of pine cones, is gaining interest due to its potential health benefits and unique bioactive components. The pine cone vinegar using the traditional fermentation method was analyzed for its chemical properties, including pH, titratable acidity, total soluble solids, and phenolic content. Various properties, such as aroma volatiles, organic acids, and total phenols related to pine cone vinegars, were also revealed. Ethyl acetate, acetic acid, isobutyl acetate, 2‐methylbutyl acetate, 4‐terpineol, alpha‐terpineol, verbenone, and 2‐phenethyl acetate stood out as key contributors to the aroma profile of the vinegars. Additionally, it has been determined that various organic acids such as oxalic acid, ascorbic acid, and formic acid are present in pine cone vinegar. The presence of high levels of terpenoids, acetic acid, and ascorbic acid detected in the CnB vinegar has enhanced its antioxidant properties. Additionally, these compounds are known for their antimicrobial properties. This makes pine cone vinegar effective against a wide range of bacteria, including common foodborne pathogens. Metagenomic sequencing helped evaluate the relative abundances at different bacterial and fungal compositions and their functional metabolite levels to reveal the diversity and variations within microbial populations. The findings suggest that pine cone vinegar could be utilized in various applications, including food preservation and health promotion, offering a sustainable and natural option for combating microbial infections.

## Author Contributions

**Duygu Alp‐Baltakesmez:** conceptualization (equal), formal analysis (equal), investigation (equal), methodology (equal), writing – original draft (equal), writing – original draft (equal). **Pelin Ertürkmen:** conceptualization (equal), formal analysis (equal), investigation (equal), methodology (equal), writing – original draft (equal), writing – review and editing (equal). **Özcan Bulantekin:** conceptualization (equal), formal analysis (equal), investigation (equal), methodology (equal), writing – original draft (equal), writing – review and editing (equal).

## Ethics Statement

The authors have nothing to report.

## Conflicts of Interest

The authors declare no conflicts of interest.

## Data Availability

The data used to support the conclusions of this research are accessible from the corresponding author upon reasonable request.
